# The Safety and Effectiveness of the Prostar XL Closure Device Compared to Open Groin Cutdown for Endovascular Aneurysm Repair

**DOI:** 10.1177/15385744231180663

**Published:** 2023-06-05

**Authors:** Tilda Hahl, Rosa Karvonen, Ilkka Uurto, Sara Protto, Velipekka Suominen

**Affiliations:** 1 Department of Vascular Surgery, 60670 Tampere University Hospital, Tampere, Finland; 2 7840 Tampere University, Tampere, Finland

**Keywords:** endovascular aneurysm repair, abdominal aortic aneurysm, percutaneous closure, prostar XL, femoral access

## Abstract

**Objective:**

The aim of this study is to compare the outcomes of percutaneous femoral closure with the Prostar XL for endovascular aneurysm repair (EVAR) to those of open femoral cutdown, and to evaluate factors which may predict the failure of percutaneous closure.

**Methods:**

Patients undergoing endovascular aneurysm repair for an infrarenal abdominal aortic aneurysm between 2005 and 2013 were included. Patient characteristics, anatomic femoral artery measurements, and postoperative complications were recorded retrospectively. Operator experience was defined with a cut-off point of >30 Prostar XL closures performed. Comparisons were made per access site.

**Results:**

A total of 443 access sites were included, with percutaneous closure used in 257 cases (58.0%) and open cutdown in 186 cases (42.0%). The complication rate was 2.7% for the percutaneous and 4.3% for the open cutdown group (*P* = .482). No significant differences between groups were found with respect to 30-day mortality, wound infections, thrombosis, seromas, or bleeding complications. Fourteen failures (5.4%) of percutaneous closure occurred. The success rates were similar for experienced and unexperienced operators (94.2% vs 95.5%, *P* = .768). Renal insufficiency was more common in the failed than in the successful percutaneous closure group (64.3% vs 24.7%, *P* = .003). Common femoral artery calcification or diameter, BMI, sheath size, or operator experience did not predict failure. No further complications were seen in follow-up CT at 1-3 years postoperatively.

**Conclusion:**

The use of the Prostar XL is safe compared to open cutdown. The success rate is 94.6%. Operator experience, sheath size, obesity, or femoral artery diameter or calcification do not appear to predict a failure of percutaneous closure. Complications seem to occur perioperatively, and late complications are rare.

## Introduction

Endovascular aneurysm repair (EVAR) has enabled faster recovery after treatment of an abdominal aortic aneurysm (AAA) compared to conventional open surgery. The common femoral artery (CFA) is traditionally exposed for EVAR by means of open cutdown. While low perioperative morbidity is one of the major advantages of EVAR, open CFA exposure is associated with high rates of wound-related problems.^1,2^ Therefore, percutaneous access for performing EVAR was introduced to offer a more minimally invasive approach to obtaining hemostasis of the groin access site and, consequently, to reduce wound-related morbidity.

A percutaneous closure system is used for the closure of the puncture site and is usually deployed before the insertion of the sheath, and the hemostatic sutures are tied at the end of the procedure. The potential advantages of percutaneous closure include a reduction in wound complications, such as post-operative seromas, wound dehiscence, and infections,^3^ as well as a shorter hospital stay,^4,5^ reduced postoperative pain,^6,7^ and a shorter operation time.^6,8,9^ However, higher morbidity after percutaneous CFA closure compared to open exposure has also been reported.^10^ The available literature shows technical success rates for different percutaneous closure systems ranging from 92% to 100%.^9,11^ Some studies have further reported predictors of technical failure, including femoral artery calcification, operator experience, and sheath size.^12,13^ Opposite results have also been reported, however, with no effects of patient characteristics on success rate.^13,14^

This study aims to review the use of percutaneous access and closure using the Prostar XL (Abbott Vascular, Santa Clara, CA, USA) device at a single institution. Prostar XL was introduced in our institution during the study period. The aim is to study perioperative and long-term complication rates compared to other closure methods, to evaluate the learning curve related to the successful use of the Prostar XL, and to analyze whether there are factors that predict technical failure.

## Material and Methods

Patients undergoing EVAR for an infrarenal AAA between January 2005 and December 2013 at a single academic institution were identified from a prospectively maintained database. Follow-up data were collected retrospectively from electronic medical records until December 31, 2016.

Patients were eligible for EVAR if they had an AAA diameter of >5.5 cm (male) or >5.0 (female), or an AAA with a rapidly increasing sac (>1 cm per year or >5 mm over a 6-month period). Ruptured aneurysm cases were excluded, but otherwise urgently managed patients with symptomatic or massive aneurysms were included.

All procedures were performed by the same group of vascular surgeons and interventional radiologists working at a single institution in a fully equipped operating room with fluoroscopic guidance. Iopromide was used as contrast medium, and patients were treated under spinal, local, or general anesthesia.

Baseline demographic data were recorded, including sex, age, body mass index, and history of smoking (never, current, ex-smoker). Comorbidities were obtained. Diabetes, hypertension, and dyslipidemia were identified when a patient was undergoing active medical treatment or diet modification. Cerebrovascular disease was defined as a history of stroke, transient ischemic attack, or cerebral hemorrhage. Coronary artery disease was identified as a history of myocardial infarction, an abnormal finding in a coronary angiogram, or angina pectoris symptoms. A history of peripheral artery revascularization was defined as peripheral artery disease. Obstructive pulmonary disease, a history of pulmonary cancer, or another pulmonary disease remarkably affecting the pulmonary function studies was recorded. Current anticoagulation therapy was recorded. Intraoperative details included the arterial closure method, device type, sheath size, and operative time.

The endografts used were the Zenith (Cook, Bloomington, IN, USA), Endurant (Medtronic, Minneapolis, MN, USA), and Excluder (W.L. Gore, Flagstaff, AZ, USA). The only percutaneous closure device included in the study was the Prostar XL (Abbott Vascular, Santa Clara, CA, USA), which is indicated for closing the common femoral artery access siteafter large introducers.

Preoperative computed tomography angiography (CTA) scans were reviewed retrospectively. The anatomic measurements included CFA diameter, anterior calcification (none, <50% circumferential anterior calcification, or ≥50% circumferential anterior calcification), and distance from skin to the CFA.

All analyses were performed per access site. In the case of a failed percutaneous closure, the access site was analyzed in an intention-to-treat group.

Groin complications were identified from documented clinical or radiological (duplex ultrasonography or CT) data. Only clinically relevant complications were determined, including any groin-related complications requiring a reoperation, wound complications requiring antibiotic or local wound therapy, seromas requiring puncture or another procedure, and pseudoaneurysms treated with a thrombin injection.

Patients with missing or low-quality preoperative CTA scans were excluded, as were those whose preoperative CTA scan did not extend down to the CFA. Patients undergoing uni-iliac endografting and femoro-femoral cross-over bypass were excluded, as were patients needing CFA endarterectomy or some other femoral artery reconstruction simultaneously. Groin punctures closed with compression or with a percutaneous closure system other than the Prostar XL were also excluded.

Operator experience was defined with a cut-off point of >30 closures performed with the Prostar XL. A failure was defined as a need for an early conversion due to bleeding or late procedures to treat a pseudoaneurysm at the access site. If necessary, manual compression after any closure method was allowed.

The imaging protocol for radiographic follow-up consisted of a CT scan within approximately 30 days postoperatively, duplex ultrasound at 1 year, a CTA scan at 2 years, and duplex ultrasound yearly thereafter. In addition to duplex ultrasound, CT was obtained if there was evidence of an endoleak or sac enlargement. The “early” and “late” follow-up CTs were reviewed. A CTA was eligible as an early follow-up CTA when taken within 3 months postoperatively, and as a late follow-up CTA, when taken 1-3 years postoperatively.

### Data Analysis

Data were expressed as medians and quartiles or means and standard deviations. Categorical variables were compared with Pearson’s Chi-squared test or Fisher’s exact test. Comparisons between the 2 groups were performed with the Mann-Whitney *U* test for continuous variables. All *P* values were two-sided, with *P* values of less than .05 regarded as indicative of statistical significance. All statistical analyses were conducted with SPSS software version 28.

This study is a retrospective study of prospectively collected registry data, and Ethics Committee or Institutional Review Board approval was not required. For the same reason, informed consent for participants was not required.

## Results

A total of 362 patients underwent an EVAR procedure for a non-ruptured AAA during the study period, 87 of whom did not have an adequate preoperative CT available and were therefore excluded. Of the remaining 550 groins, 47 were excluded due to a femoro-femoral crossover, CFA endarterectomy, or femoral aneurysm reconstruction. Sixty groins were compressed and excluded. Thus, the inclusion criteria were met by 443 access sites (in 258 patients), and percutaneous closure was used in 257 (58.0%) and open cutdown in 186 cases (42.0%). The number of percutaneously closed access sites increased during the study period (*P* < .001).

Patients who were treated with a Zenith endograft were more likely to have an open cutdown than percutaneous closure (59.1% vs 40.9%, *P* < .001), while those who were treated with an Endurant or Excluder endograft were more likely to have a percutaneous closure than an open cutdown (73.7% vs 26.3%, *P* < .001; 72.9% vs 27.1% *P* < .001). Large sheath size (≥18 F) was used more frequently in the open cutdown group than in the percutaneous group (89.8% vs 40.5%, *P* < .001). The operation time was shorter for the percutaneous closure group than for the cutdown group (median 102 min vs 114 min, *P* < .001), as was the hospital stay (mean 3.0 d vs 3.5 d, *P* = .004). CFA diameter was smaller in patients receiving percutaneous closure compared to those with open cutdown (median 10.5 mm vs 11.1 mm, *P* < .001). Otherwise, no significant differences between groups were found with respect to baseline characteristics. (Table 1.)

**Table 1.** Baseline Characteristics of the Percutaneous Closure and Open Cutdown Groups.

	Percutaneous	Open	*P*
	n = 257	n = 186	
Age (y), median	76.3	77.9	.192
Q1; Q3	71.3; 82.1	72.6; 82.0	
Male	86.8	89.2	.465
Urgent	5.4	4.8	.831
Anticoagulation	21.8	25.3	.426
Diabetes	21.0	16.1	.220
Dyslipidemia	46.7	46.8	.986
Hypertension	65.0	62.9	.689
Coronary disease	44.4	53.8	.054
Cerebrovascular disease	14.8	19.4	.245
Pulmonal	19.5	22.6	.477
Renal insufficiency	26.8	26.3	.914
Peripheral artery disease	5.4	5.4	.974
Smoking			
Current	32.1	27.0	.473
Ex-smoker	41.4	49.0	
Never	26.5	24.0	
Body mass index	26.2	26.1	.892
Q1; Q3	23.7; 29.1	24.2; 29.1	
CFA calcification			
No	58.0	60.2	.587
Yes, <50%	30.0	25.8	
≥50%	12.1	14.0	
CFA diameter (mm), median	10.5	11.1	**< .001**
Q1; Q3	9.7; 12.0	10.0; 12.7	
Distance from skin to CFA (mm), median	28.3	30.0	.453
Q1; Q3	21.0; 38.5	21.3; 38.7	
Sheath size			
12 - 16 F	59.5	10.2	**< .001**
18 - 20 F	40.5	89.8	
Operation time (min), median	102	114	**< .001**
Q1; Q3	84; 120	96; 138	
Length of hospitalisation (d), mean +/− SD	3.0 +/−1.9	3.5 +/−1.7	**.004**

Note: Values are presented as percentages unless stated otherwise. CFA = common femoral artery.

The 30-day mortality was similar in both groups. Seven access sites required an early reoperation, which occurred at 0-29 days postoperatively, with a median of .5 days for the percutaneous closure group and 4 days for the open cutdown group (*P* = .400). Any clinically relevant complication occurred at 7 access sites (2.7%) in the percutaneous group and at 8 access sites (4.3%) in the open cutdown group, but this difference was not statistically significant (*P* = .429). Two access sites in the percutaneous group developed 2 different complications. No significant differences between the groups were found according to the complication rates (Table 2).

**Table 2.** Comparison of Complications Between the Percutaneous Closure and Open Cutdown Groups.

	Percutaneous	Open	*P*
	n = 257	n = 186	
30d mortality	3 (1.2)	1 (.5)	.642
Reoperation	4 (1.6)	3 (1.6)	.962
Any clinically significant complication	7 (2.7)	8 (4.3)	.429
Wound infection	1 (.4)	5 (2.7)	.087
Thrombosis	2 (.4)	0 (.0)	.512
Seroma	0 (.0)	1 (.5)	.420
Any bleeding/pseudoaneurysm	6 (2.3)	2 (1.1)	.477
Pseudoaneurysm and reoperation	0 (.0)	0 (.0)	
Pseudoaneurysm and thrombin	3 (1.2)	0 (.0)	
Acute bleeding	3 (1.2)	0 (.0)	
Hematoma evacuation	0 (.0)	2 (1.1)	

Note: Values are presented as number (%).

Early follow-up CT was done for 96.6% of the access sites, but the scan extended down to the femoral vessels for only 29.6%. The early CT scan was performed 0-94 days postoperatively. Two early follow-up CTs were taken during a bleeding complication following a reoperation, but no other significant complications were seen in these CT scans. Late follow-up CTs (taken at 367-1091 days postoperatively) were done for 63.5% of the access sites, but the scan was diagnostic for 40.6% of the groins. No significant complications were seen in these late CT scans.

### Failed Percutaneous Closures

Fourteen failures (5.4%) of the percutaneous closure were recorded, 8 of which (3.1%) occurred during the deployment of the device or at the end of the operation and were converted to an open cutdown. The remaining 6 failures (2.3%) were noticed postoperatively – 3 of these required a reoperation, while 3 pseudoaneurysms were treated with a thrombin injection. In addition, 1 patient in the percutaneous closure group needed an embolectomy for a thrombosis which most likely occurred due to prolonged compression after inadequate hemostasis with a percutaneous closure device. One of the failures was the 10th procedure performed by the operator with Prostar XL, while 1 was the operator’s 14th and 1 the operator’s twenty-second Prostar XL procedure. The other failures occurred beyond the cut-off point of 30. Details of the failed percutaneous closures are listed in Table 3. The success rates for unexperienced and experienced operators were similar (95.5% vs 94.2%, *P* = .768). Furthermore, when comparing the failed and successful percutaneous closure groups, there was no difference in the number of unexperienced operators (under the cut-off point; 21.4% vs 26.3%, *P* = .481). Renal insufficiency was statistically more common in the failure group (64.3% vs 24.7%, *P* = .003). Diabetes was also more common in the failure group, but this was not statistically significant (42.9% vs 19.8%, *P* = .083). No differences in sheath size, femoral calcification or diameter, distance from skin to the CFA, BMI, or any other baseline characteristics were found between the groups (Table 4).

**Table 3.** Details of the Failed Percutaneous Closures.

Patient No.	Operator Experience(Ordinal Number)	Possible Reason(s) for Failure	Sheath Size (F)	Treatment
1	10	Suture pulled through artery	14	Conversion
2	14	Age 88 years, major CFA calcification	18	Conversion
3	22	Technical failure	12	Conversion
4	68	BMI 39, distance from skin to CFA over 100 mm	16	Conversion
		Minor calcification		
		Venous puncture		
5	80	No explanation	18	Conversion
6	140	Distance from skin to CFA 40 mm	18	Conversion
		Minor calcification, anticoagulation		
7	145	Technical failure	20	Conversion
8	152	Anticoagulation	18	Conversion
9	36	Minor calcification	14	Open repair
10	41	Suture pulled through artery	20	Open repair
11	43	Distance from skin to CFA 45 mm	14	Thrombin treatment
12	49	Age 87 years	18	Thrombin treatment
13	97	Distance from skin to CFA 45 mm	14	Thrombin treatment
		Minor CFA calcification, anticoagulation		
14	109	Urgent operation, major calcification	18	Open repair
		Suture pulled through artery, iliac thrombosis during reoperation		

Note: CFA = common femoral artery.

**Table 4.** Comparison Between the Failed and Successful Percutaneous Closures.

	Failed	Successful	*P* value
	n = 14	n = 243	
Age >85 y	3 (21.4)	23 (9.5)	.158
Male	12 (85.7)	211 (86.8)	.576
Urgent	1 (7.1)	13 (5.3)	.553
Endoprosthesis			
Zenith	4 (28.6)	81 (33.3)	.943
Endurant	4 (28.6)	66 (27.2)	
Excluder	6 (42.9)	96 (39.5)	
BMI ≥30	2 (14.3)	54 (23.3)	.742
Sheath size ≥18	8 (57.1)	96 (39.5)	.263
Anticoagulation	5 (35.7)	51 (21.0)	.194
Diabetes	6 (42.9)	48 (19.8)	.083
Dyslipidemia	8 (57.1)	112 (46.1)	.583
Hypertension	8 (57.1)	159 (65.4)	.570
Coronary disease	8 (57.1)	106 (43.6)	.410
Cerebrovascular disease	3 (21.4)	35 (14.4)	.442
Pulmonal	2 (14.3)	48 (19.8)	.465
Renal insufficiency	9 (64.3)	60 (24.7)	**.003**
Peripheral artery disease	0 (.0)	14 (5.8)	.689
CFA diameter <9 mm	0 (.0)	33 (13.6)	.227
Distance from skin to CFA ≥40 mm	4 (28.6)	55 (22.6)	.533
Anterior CFA calcification			
1%-50%	5 (35.7)	72 (29.6)	.724
≥50%	2 (14.3)	29 (11.9)	
Unexperienced operators	3 (21.4)	64 (26.3)	.481

Note: Values are presented as number (%). BMI = body mass index, CFA = common femoral artery.

## Discussion

According to the present study, the complication rates did not differ significantly between the access site closure methods. The success rate of percutaneous closure was 94.6%. Renal insufficiency was more common in the failed than in the successful percutaneous closure group. Otherwise, no factors predicting the failure of percutaneous closure were found.

The operation time was shorter for the percutaneous closure group compared to the open cutdown group (median 102 min vs 114 min, *P* < .001). Due to the retrospective nature of this study, the specific duration of access site closure could not be determined. Hospital stays were also shorter in the percutaneous group. The percutaneous devices are expensive, and potential savings in the form of a reduced operation time and duration of hospitalization could offset the cost of the devices. The analysis was performed per access site, and a patient could have 2 different closure methods used on them, which most likely has an impact on operation time and duration of hospitalization. In the literature, percutaneous closure is associated with reduced procedure and access times^5,6,8,9^ as well as shorter hospital stays^15^ compared to open cutdown. However, 1 meta-analysis on the topic did not find differences in the duration of surgery or length of hospitalization between percutaneous and surgical access.^3^

In the current cohort, open cutdown was used more often when large sheath sizes (larger endografts) were required. Vascular access for the main body of the endoprosthesis was usually achieved by means of open cutdown during the introduction period of the Prostar XL closure device at our clinic, which explains the difference. Despite this, the effect of a large sheath size on the failure of percutaneous closure was nonsignificant. A trend towards a more frequent use of percutaneous closure occurred over the study period. At same time, the trends of using either Zenith, Endurant, or Excluder endografts varied at our clinic, which explains the difference in endografts between the groups.

Only a limited number of randomized trials comparing percutaneous closure and open cutdown are found in the literature. Nelson et al. included 151 patients and found a reduced risk of major access-related complications, such as vascular injury, lower extremity ischemia, bleeding, and nerve injury, when comparing the ProGlide (Abbott Vascular, Santa Clara, CA, USA) percutaneous closure device with open cutdown, but they noticed no difference between the Prostar XL and open cutdown.^16^ Another trial with 50 patients showed fewer complications, including femoral artery occlusions, surgical wound revisions, leg edemas, hematomas, and seromas, after percutaneous closure than after open cutdown.^6^ However, 2 randomized studies have found similar complication rates after open cutdown and percutaneous closure.^5,7^ 1 of these studies included 274 access sites and the other only 55 access sites. Fewer access-related complications (risk ratio [RR] .47, 95% confidence interval [CI] .28-.78, *P* = .004) were also reported in a systematic review including 22 studies.^9^ Another meta-analysis of 17 studies and 7889 vascular access sites found fewer postoperative seromas (odds ratio [OR] .15, 95% CI .06-.35), less wound dehiscence (OR .14, 95% CI .03-.78), and fewer surgical site infections (OR .38, 95% CI .23-.63) after percutaneous closure than after open cutdown.^3^ The same meta-analysis reported more pseudoaneurysms after percutaneous closure (OR 3.83, 95% CI 1.55-9.44). In the present study, any complication occurred in 2.7% of the percutaneous closure group, while the complication rate for open cutdown was 4.3%. This was, however, not statistically significant. The overall incidence of complications in this study population was small, which may explain why differences between groups failed to reach statistical significance.

Complications occurred at a median of 1 day and at a range of 0-43 days postoperatively. Follow-up CTs did not reveal any further complications, which is consistent with previous studies, with late complication rates of 0%-2%.^17,18^ Although adequate post-operative CT scans were available for only 29.6% of patients, all patients had a clinical examination approximately at 30 days postoperatively and at 1 and 2 years. In addition to clinical examination, ultrasonography was done at the follow-up visits. Therefore, it is unlikely that a relevant complication would have been missed.

The overall success rate was 94.6%, which is consistent with previous reports, with technical success rates of 92%-96.9%.^4,8,18,19^ The success rate more specifically for Prostar XL has been reported to be 88.8%-96.1%.^12,13,16^ Only renal insufficiency was a risk factor for a failed percutaneous closure. This association may be only coincidental, though statistically significant. In addition, a more minimally invasive technique may have been recommended for comorbid patients, indicating that the significance of this finding is not clinically relevant. The assumption was that CFA calcification and diameter, distance from skin to CFA, BMI, current anticoagulation, or sheath size could predicted failure, which could not be proved. The risk factors found in the literature include femoral artery calcification^12,18^ and sheath size.^12,13^ Similarly to the results of the present study, obesity has not been reported as a risk factor.^12,13,18^ At the same time, 1 previous study found no obvious risk factors for groin complications after percutaneous closure.^14^

Another assumption that could not be confirmed in the present study was the possible association between a learning curve and failure. The used cut-off point of >30 closures performed with the Prostar XL was based on previous studies about the topic.^5,12^ A strong correlation with operator experience was reported in a study with 903 percutaneous accesses with same cut-off point (OR 43.2, 95% CI 9.8-189.0, *P* < .001).^12^ In a randomized trial by Larzon et al, the success rate of Prostar was 93% for the proficiency-level group (≥60 procedures performed), while it was only 70% for the basic-level group (≥15 procedures performed).^20^ The success rate of the basic-level group differs greatly from the 95.5% for unexperienced operators in the present study. Compression following percutaneous closure was not defined as a failure, and, unfortunately, due to the study design, it is not possible to determine whether it is more common among unexperienced operators. Furthermore, the number of closure devices used per access site was not included, and an unexperienced operator may have more frequently used more than 2 devices for successful hemostasis.

Prostar XL was the only closure device included in the present study. However, other percutaneous closure devices are commercially available. A systematic review article including 2909 patients showed that Prostar XL is associated with greater risk of bleeding compared to ProGlide (RR 1.82, 95% CI 1.47-2.24, *P* < .001).^21^ In addition, life-threatening bleeding complications occurred more frequently in patients treated with Prostar XL.

Several limitations of the present study should be acknowledged. Firstly, this is a non-randomized retrospective study, and the results may be affected by the limitations regarding data selection. After the introduction of percutaneous closure at our clinic, only some patients were treated with this technique. The number of these patients increased during the study period, with no established protocol on when to offer percutaneous closure. Over the years, mainly after 2011, most patients were treated with the Prostar XL. In addition, preoperative management with a preoperative CT scan allowed operators to select percutaneous closure for only patients with a suitable anatomy. Data on a possible groin scar were not reliable and could not be included in the analysis. Many patients were excluded because of an inadequate preoperative CTA, reducing the study population. Finally, the number of adverse events, including groin complications and failures of percutaneous closures, was small, which limits statistical power to a great extent. On the other hand, the present study demonstrates that the complications and failures of the percutaneous closure are rare.

In conclusion, the data presented suggest that the use of the Prostar XL is safe, with a success rate of 94.6%. The use of the device also seems to be feasible among unexperienced operators. Sheath size, obesity, or femoral artery diameter or calcification does not appear to influence the outcome. In addition, complications mainly occur perioperatively, and late complications are rare.
